# Correlation of macular sensitivity measures and visual acuity to vision-related quality of life in patients with age-related macular degeneration

**DOI:** 10.1186/s12886-021-01901-x

**Published:** 2021-03-23

**Authors:** Thomas Richard Johansen Forshaw, Alexandra Kalia Parpounas, Torben Lykke Sørensen

**Affiliations:** 1 Department of Ophthalmology, Zealand University Hospital, Vestermarksvej 23, DK-4000 Roskilde, Denmark; 2 Faculty of Health and Medical Sciences, University of Copenhagen, Copenhagen, Denmark

**Keywords:** Age-related macular degeneration, Early treatment diabetic retinopathy study, Macular sensitivity, Microperimetry, Visual acuity, Vision-related quality of life, Visual function questionnaire 39

## Abstract

**Background:**

Visual acuity is commonly used as a functional outcome measure in patients with age-related macular degeneration (AMD), despite having a weak correlation with self-perceived visual quality of life. Microperimetry is a useful method of detecting loss of macular function. We wanted to investigate the relationship between these two objective visual outcome measures and subjective vision-related quality of life, finding out which objective measure is more patient-relevant.

**Methods:**

Fifty-one consecutive patients with AMD were recruited to the study. Participants were required to complete the Visual Function Questionnaire 39, the Early Treatment Diabetic Retinopathy Study visual acuity examination and a microperimetry assessment using the Micro Perimeter 3. One patient withdrew consent and seven patients dropped out due to cooperation difficulties under microperimetry. Forty-three patients with AMD were included in the study: twenty-eight patients with late AMD (exudative AMD) and fifteen patients with early (non-exudative) AMD. The right eye was included as standard, as was the eye with the best-corrected visual acuity.

**Results:**

There was a higher correlation between vision-related quality of life and macular sensitivity (r = 0.458; *p* = 0.014) than between vision-related quality of life and visual acuity (r = 0.446; *p* = 0.018) in patients with late AMD. There was a positive correlation between vision-related quality of life and macular sensitivity in patients with early AMD (r = 0.542; *p* = 0.037) while the correlation between vision-related quality of life and visual acuity in these patients was not statistically significant. Composite score (r = 0.469; *p* = 0.012) correlated highest with the nasal outer macular sub-region and near-distance activities score (r = 0.652; *p* < 0.001) correlated highest with the nasal inner macular sub-region in patients with late AMD. Correlations between composite score and macular sub-regions in patients with early AMD were not significant, but near-distance activities score correlated with the nasal outer macular sub-region in these patients (r = 0.469; *p* = 0.012).

**Conclusions:**

Macular sensitivity as measured using microperimetry correlates with vision-related quality of life in early AMD and in late AMD, showing it to be a patient-relevant outcome measure. Furthermore, the nasal sub-regions of the macula appear to be preferred retinal loci in patients with AMD.

(338 words)

**Supplementary Information:**

The online version contains supplementary material available at 10.1186/s12886-021-01901-x.

## Background

Age-related macular degeneration (AMD) is the leading cause of vision loss in the developed world [1]. The disease impacts vision-related quality of life, as well as having a substantial medical cost [2–5]. In clinical practice, monitoring of AMD is most often reliant on visual acuity and optical coherence tomography (OCT) even though it is known that both these measures have a relatively weak correlation with measures of patient self-reporting such as the Visual Function Questionnaire (VFQ) [3, 6]. Outcome measures that are more patient-relevant are being sought after in clinical trials, but since patient relevance can be difficult to quantify, it is important to identify the objective measure of visual function most closely aligned with subjective experience.

Microperimetry is a non-invasive measure of macular sensitivity that can provide valuable information about visual dysfunction in patients with AMD, including location and size of lesions in the macula and how they affect fixation [3, 4, 7–9]. Studies involving patients with diabetic retinopathy have suggested that microperimetry could be a better objective measure in quantifying visual function than visual acuity [10, 11], a claim supported by studies investigating AMD [12, 13]. We wanted to investigate the relationship between vision-related quality of life measured using VFQ and objective measures of visual function, namely, visual acuity and microperimetry. In this, the first study of its kind, our primary aim was to find the objective measure that is most patient-relevant in patients with AMD.

Microperimetry can measure the function of different areas of the macula and studies have shown differences in the way in which patients with AMD fixate compared with individuals without vitreoretinal disease [14, 15]. As an additional aim, we wanted to investigate if different areas of the macula correlated better with VFQ, and if certain areas of the macula are more important than others in patients with AMD.

## Methods

### Study design and participants

Based on previous studies [16–18] and with a given significance value of 0.05 and a power of 80%, we calculated that thirty-four patients with AMD were required to undergo retinal function testing. Due to the risk of dropout, the minimum number required was increased to forty. From October 2017 to March 2019 fifty-one consecutive patients with AMD were recruited from the outpatient department of Zealand University Hospital.

A consultant ophthalmologist performed a full ocular examination at the start of the study. Although patients had been diagnosed with AMD in the years prior to the study, fundus examination and OCT were used to confirm the diagnosis. No patients recently diagnosed with AMD were included in this study. Patients with a vitreoretinal pathology other than AMD, glaucoma with visual field defect, amblyopia or cognitive deficit were excluded from the study. One patient withdrew consent and seven patients dropped out from the study for failing to complete the microperimetry assessment. Of these seven, five were unable to fixate on the target during the investigation, one fell asleep and one had a stiff neck and found the chin rest to be too uncomfortable. Forty-three patients with AMD were therefore included in the study. These were: twenty-eight patients with late AMD (exudative AMD) and fifteen patients with early (non-exudative) AMD. Patients with geographic atrophy were not included in this study so there would not be any visual acuity disparity within the late AMD subgroup. Although this phenotype is a late stage of the disease, it is not uncommon for these patients to have so-called foveal sparing allowing them to maintain good visual acuity, unlike patients with exudative AMD.

Additionally, for the purposes of investigating how patients with AMD differ in the way in which they fixate compared with individuals with healthy retina, we included thirty-two individuals without vitreoretinal disease as a control group. These individuals were also recruited from the outpatient department of our university teaching hospital.

### Measurement of vision-related quality of life

Vision-related quality of life was measured using the National Eye Institute Visual Function Questionnaire-39 (VFQ). Several instruments for measuring vision-related quality of life exist, but we chose the National Eye Institute Visual Function Questionnaire-39 (VFQ) because our patients are Danish speakers and a Danish language version of this questionnaire exists that is validated for use in patients with AMD [19].

The VFQ lasts approximately twenty minutes and consists of thirty-nine items concerning the self-reported visual health status of an individual. The overall VFQ composite score and the near activities sub-score were included in our analysis of results. VFQ investigations were performed by one of the authors, T.R.J.F., preferably face-to-face, or by telephone. Interviews were conducted by telephone when patient limitations such as advanced age and disability did not allow completion of both the questionnaire and the microperimetry investigation during the same visit and when geographical distance and transport costs rendered a second visit unfeasible. Telephone interviews were permitted to provide flexibility in this regard and were conducted within 1 week of the patient visit. All interviews were conducted in a designated research room and a clear interview guide was used. Interviews were one-to-one unless a study participant felt it necessary to have their next of kin present. In such cases, the investigator made a note of this both during and after the interview.

### Visual acuity

Visual acuity was examined according to departmental guidelines using the Early Treatment Diabetic Retinopathy (ETDRS) method. Some participants only had best-corrected visual acuity (BCVA) reported in Snellen; these values were converted to ETDRS before performing statistical analysis. Snellen to ETDRS conversion was performed for five participants in the AMD group and twelve participants in the group without vitreoretinal disease.

### Microperimetry

Microperimetry is a useful method of detecting loss of macular function in patients with AMD [5]. All microperimetry investigations were performed by one of the authors, T.R.J.F. using a single Nidek Micro Perimeter 3 (MP-3) (Nidek Co., Ltd. Gamagori, Japan) according to the operator instructions. The assessments were conducted in a dark room while the contralateral eye was patched. In the case of significant eye movements, which occurred often due to poor fixation in patients with AMD, the test was paused automatically. Moreover, the test was paused if the patient needed a break. The test could then resume after successful realignment of the study eye.

Microperimetry measures differential light sensitivity (DLS) in decibels at thirty-three different points in the region of the macula. DLS defines as the “minimal luminance of a white spot stimulus superimposed on a white background of uniform luminance necessary to perceive the stimulus” [7]. We calculated the mean overall macular sensitivity for each eye using 95% confidence intervals and we performed local analysis of macular sensitivity by macular sub-region.

**Fig. 1:**
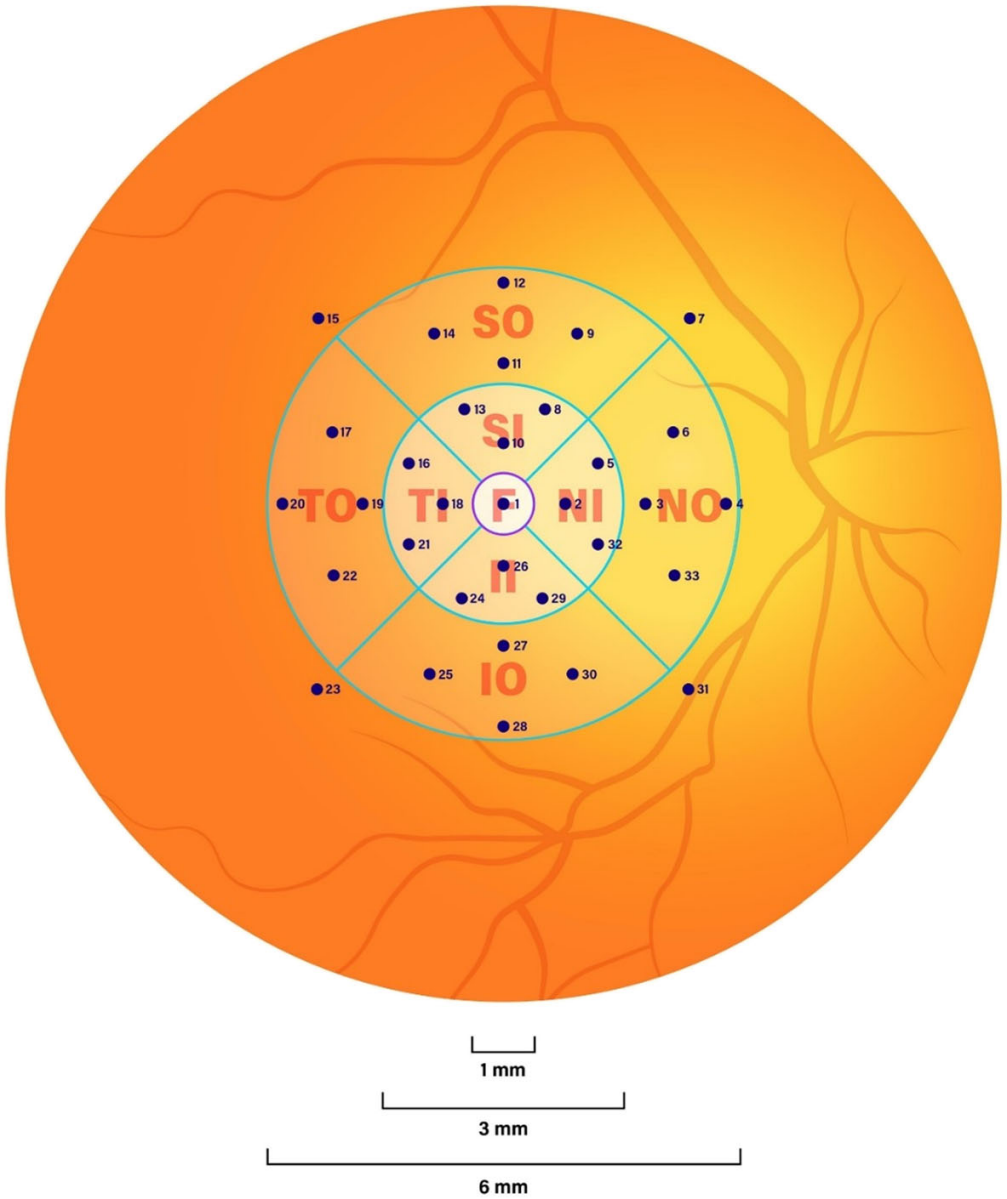
Diagram of a fundus image with superimposed Early Treatment of Diabetic Retinopathy Study macular grid. Numbered dots show the locations of the individual retinal sensitivity measurements or differential light sensitivity points. Note that four differential light sensitivity points numbered 7, 15, 23 and 31 lie outside the Early Treatment of Diabetic Retinopathy Study grid

To study the individual macular sub-regions, we superimposed a standard ETDRS grid onto a fundus image obtained by microperimetry. The ETDRS macular grid [20] is a tool that allows the macula to be divided into sub-regions when projected onto a fundus image [21, 22]. The macular subfields defined by the ETDRS grid are: the fovea, superior inner, temporal inner, inferior inner, nasal inner, superior outer, temporal outer, inferior outer, and nasal outer [7]. Each DLS point corresponds with a number that determines which ETDRS sub-region it falls into. *(*Fig. 1*).*

Using Microsoft Excel (Microsoft Co. Redmond, WA), we calculated the mean of the DLS points within each macular sub-region with 95% confidence intervals. The fovea was comprised of a single, central DLS point; the four inner sub-regions each contained three DLS points and the four outer sub-regions each contained four DLS points. Four DLS points fell outside of the superimposed ETDRS grid and were therefore excluded from local analysis but were included in the overall mean macular sensitivity calculation.

Finally, we correlated data from the overall macula and the individual macular subfields with visual function as defined by the VFQ.

### Data analysis

Results were analysed using two different methods: standard eye analysis and best eye analysis. (Table 1) Standard eye analysis used data from the right eye, as far as possible. Exceptions to this rule were: cases in which a patient had clinical evidence of AMD only in the left eye on ophthalmic examination and/or a missing or incomplete right eye microperimetry investigation. A patient’s best eye was defined as the eye with highest BCVA.

**Table 1:** Comparison between best eye analysis and standard eye analysis data

	Best Eye Analysis^a^						Standard Eye Analysis^b^					
	**Early AMD Group**	**Late AMD Group**	**Healthy Retina Group**	***p*** **values**			**Early AMD Group**	**Late AMD Group**	**Healthy Retina Group**	**p values**		
				**p**^**1**^	**p**^**2**^	**p**^**3**^				**p**^**1**^	**p**^**2**^	**p**^**3**^
Visual acuity (ETDRS)^c^	74.3 Std. Error: 2.6 (6/9.5 Snellen)	68.3 Std. Error: 2.0 (6/15 Snellen)	77.4 Std. Error 1.9 (6/9 Snellen)	1.0	0.006	0.186	72.1 Std. Error: 3.2 (6/12 Snellen)	63.7 Std. Error: 2.5 (6/15 Snellen)	72.7 Std. Error: 2.4 (6/12 Snellen)	1.0	0.04	0.12
Total macular sensitivity (dB)	21.9 IQR: 8.8	18 IQR: 10	22.61 IQR: 7	0.059	< 0.001	0.016	21.9 IQR: 8.8	15.2 IQR: 13	22.61 IQR: 7	0.151	< 0.001	0.014

^a^Eye with best-corrected visual acuity used

^b^Right eye used as standard

^c^Analysis of Covariance (ANCOVA) post-hoc tests

*AMD* age-related macular degeneration; *dB* decibels; *ETDRS* Early Treatment Diabetic Retinopathy Study; *IQR* interquartile range; p^1^ = early AMD compared with healthy retina; p^2^ = late AMD compared with healthy retina; p^3^ = early AMD compared with late AMD

Best eye analysis was required to correlate BCVA and macular sensitivity to VFQ. Objective means of measurement of visual function do not always correlate with patients’ self-perceived visual abilities [19], but it seemed likely that the better-seeing eye would be the more important eye in terms of subjective visual function. Best eye analysis was therefore used to determine if BCVA or macular sensitivity related better to vision-related quality of life. Best eye analysis was also used to investigate the correlation between different macular sub-regions and VFQ and to show how specific areas of the macula relate to aspects of visual function. Standard eye analysis was performed to avoid selection bias when investigating the effects of AMD on macular sensitivity. We used this method to compare the different ETDRS subfields to find out which areas of the macula are more important in patients with AMD.

Data Analysis was performed using SPSS Statistical Analysis Software (IBM Corporation Armonk, NY). We used the Shapiro-Wilk test of normality to determine data distribution. In the case of normal distribution, parametric tests (Pearson’s coefficient; independent samples t-test) were used. In the absence of normal distribution, non-parametric tests were used (Spearman’s rho coefficient; Mann-Whitney U-test). A *p* value of < 0.05 was considered statistically significant.

## Results

We included fifteen patients with early AMD (mean age: 77.5 ± 7.2 years), twenty-eight patients with late AMD (mean age: 79.1 ± 5.3 years), and thirty-two individuals without vitreoretinal disease (mean age: 71.7 ± 7.8 years) as a control group. The control group was significantly younger than the group with AMD (*p* < 0.001; independent samples test) and we used linear regression to adjust for this. After adjusting for age, there was a significant difference between the groups in terms of macular sensitivity (*p* = 0.048) but the difference in visual acuity was not significant (*p* = 0.059). Demographic data is available as an additional file (see Additional file 1). Visual acuities are reported in Table 1.

**Fig. 2:**
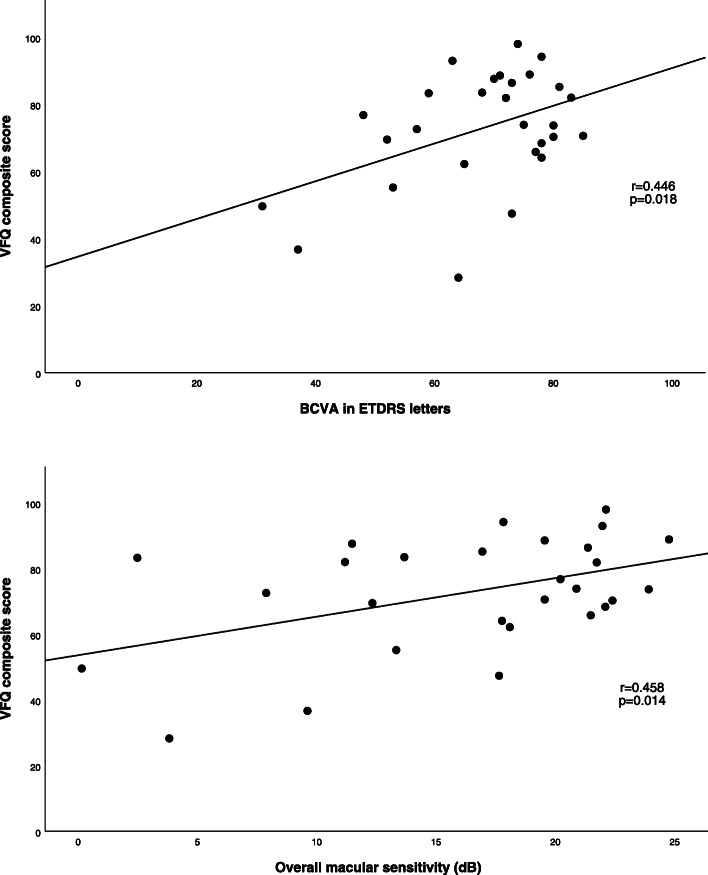
Visual Function Questionnaire and visual acuity and macular sensitivity correlations in late age-related macular degeneration

With regards the main aim of this study, there was a positive correlation between VFQ composite score and BCVA in patients with late AMD (r = 0.446; *p* = 0.018 Pearson’s Correlation), but the correlation between VFQ composite score and overall macular sensitivity in these patients was higher (r = 0.458; *p* = 0.014). (Fig. 2) There was a positive correlation between VFQ composite score and overall macular sensitivity in patients with early AMD (r = 0.542; *p* = 0.037) but the correlation between BCVA and VFQ composite score was not statistically significant. There were no significant differences between males and females in the VFQ scores and their association with visual acuity and microperimetry measures. Full correlation data for early and late AMD are shown in Table 2 and Table 3 respectively. When we tested the relationship between BCVA and macular sensitivity in patients with late AMD we found there to be a positive correlation (rho = 0.502; *p* = 0.007); however, the same correlation was not statistically significant in patients with early AMD.

**Table 2:** Visual Function Questionnaire correlated with visual acuity and macular sensitivity in early age-related macular degeneration

VFQ sub-score		Best-corrected visual acuity	Macular sensitivity
Composite	r	0.130	0.542^b^
	p value	0.644	0.037
General Vision	r	0.339	0.479
	p value	0.217	0.071
Near Activities	r	0.306	0.595^b^
	p value	0.268	0.019
Distance Activities	r	0.059	0.664^a^
	p value	0.834	0.007
Peripheral Vision	rho	−0.027	0.039
	p value	0.928	0.896
Driving	r	0.005	0.450
	p value	0.989	0.165
Social Functioning	rho	0.019	0.617^b^
	p value	0.946	0.014
Role Difficulties	rho	0.042	0.259
	p value	0.883	0.351
Dependency	rho	−0.011	0.323
	p value	0.970	0.241
Mental Health	r	0.318	0.412
	p value	0.249	0.127
Color Vision	rho	−0.091	0.048
	p value	0.747	0.864
Ocular Pain	rho	0.259	0.045
	p value	0.351	0.873
General Health	rho	0.119	0.536^b^
	p value	0.673	0.04

^a^indicates correlation is significant at the 0.01 level (2-tailed)

^b^indicates correlation is significant at the 0.05 level (2-tailed)

*VFQ* Visual Function Questionnaire

**Table 3:** Visual Function Questionnaire correlated with visual acuity and macular sensitivity in late age-related macular degeneration

VFQ sub-score		Best-corrected visual acuity	Macular sensitivity
Composite	r	0.446^b^	0.458^b^
	p value	0.018	0.014
General Vision	r	0.236	0.154
	p value	0.227	0.433
Near Activities	r	0.611^a^	0.598^a^
	p value	< 0.001	< 0.001
Distance Activities	r	0.379^b^	0.271
	p value	0.047	0.163
Peripheral Vision	rho	0.068	0.455^b^
	p value	0.729	0.015
Driving	r	0.469	0.656^a^
	p value	0.058	0.004
Social Functioning	rho	0.258	0.203
	p value	0.186	0.301
Role Difficulties	rho	0.352	0.368
	p value	0.066	0.054
Dependency	rho	0.259	0.357
	p value	0.184	0.62
Mental Health	r	0.312	0.312
	p value	0.107	0.107
Color Vision	rho	0.098	0294
	p value	0.621	0.129
Ocular Pain	rho	−0.115	0.168
	p value	0.559	0.393
General Health	rho	−0.145	0.048
	p value	0.461	0.808

^a^indicates correlation is significant at the 0.01 level (2-tailed)

^b^indicates correlation is significant at the 0.05 level (2-tailed)

*VFQ* Visual Function Questionnaire

To address the secondary purpose of this study, which was to investigate how patients with AMD differ from those with healthy retina in terms of how they preferentially recruit sub-regions of the macula, we obtained macular sensitivities measured in decibels for eyes with AMD and eyes without vitreoretinal disease. The overall macular sensitivities were 21.9 (interquartile range (IQR): 8.8) in the early AMD sub-group and 18 (IQR: 10) in the late AMD sub-group, compared with 22.61 (IQR: 7) in the healthy retina group (*p* < 0.001; Mann-Whitney U-test).

In patients with AMD and individuals without vitreoretinal disease, the area of the macula with the highest macular sensitivity was the temporal outer sub-region. The macular sensitivities in this area were: 23.0 (IQR: 7) in the early AMD sub-group and 22.8 (IQR: 8) in the late AMD sub-group compared with 23.59 (IQR: 7) in the healthy retina group, a difference that was statistically significant (*p* = 0.012; Mann-Whitney U-test). Macular sensitivity results are provided as an additional file (see Additional file 2).

When we correlated the different ETDRS subfields with VFQ, the VFQ composite score correlated highest with the nasal inner macular sub-region (rho = 0.508; *p* = 0.06) in patients with late AMD, but this correlation was not statistically significant. The second highest correlation was the nasal outer macular sub-region, (r = 0.469; *p* = 0.012) suggesting that this area of the macula is important in terms of vision-related quality of life in these patients. Correlations between composite score and macular sub-regions in patients with early AMD and individuals with healthy retina were not significant.

The VFQ near-distance activities sub-score correlated with the nasal inner macular sub-region in patients with late AMD (r = 0.652; p = < 0.001), and with the nasal outer sub-region in patients with early AMD (r = 0.469; *p* = 0.012), suggesting that the nasal macula is important to this aspect of visual functioning in patients with AMD. Correlations between near-distance activities sub-score and macular sub-regions in individuals with healthy retina were not significant. Full correlation data is available as an additional file (see Additional file 3).

## Discussion

BCVA is commonly used as an outcome measure in clinical studies and as a general measure of visual function. The need for an objective method of testing visual outcome, such as BCVA, in response to treatment is widely accepted. BCVA has a low correlation with VFQ [19]. Microperimetry has been shown to correlate significantly with visual acuity (*p* = 0.0001) [23]. Our results now suggest that microperimetry could be a superior measure of visual outcome than visual acuity because it correlates better with VFQ.

The importance of VFQ as a subjective method of vision assessment is accepted and it is acknowledged that responses may differ depending on how the questionnaire is administered [24]. Most studies that compare different modes of administration find a small or negligible impact on the results [25, 26], although one study reported that telephone administration is associated with more positive quality of life scores [27]. However, limitations due to disability should not be a factor in determining whether an individual is eligible to be interviewed, and it makes sense to provide flexibility in this regard by interviewing over the phone [28]. Nevertheless, face-to-face interviews are often preferable as this mode allows for the observation of non-verbal cues, providing a more natural type of interchange between the interviewer and the subject [29]. Responses can be influenced either over the phone or face-to-face, through inflections of the voice, gestures and facial expressions [30] and it is therefore important that an interviewer maintains the neutral tone of voice and style of delivery set out by the interview guide when administering the VFQ.

Unlike microperimetry, which uses decibels, visual acuity has no standardised measurement as distances can be measured in feet or metres. In addition, there are several different methods of visual acuity examination in clinical use [6, 31, 32], making it difficult to directly compare results from different clinics and research centres. Visual acuity measures a person’s ability to discriminate between stimuli when presented on a highly contrasted background [6]. For routine visual acuity assessment in daily clinical practice, the Snellen Visual Acuity Chart or the ETDRS Chart is generally used [32].

Previous studies recommend the ETDRS method of visual acuity measurement in patients with AMD because it has better accuracy and reproducibility than Snellen, particularly in patients with advanced disease [6, 31, 33]. This is especially relevant as visual acuity may not be affected in patients with AMD until the disease has progressed into the late stage [3, 9, 12]. The ETDRS chart measures visual acuity from a distance of four metres, so a specially adapted room is required, which is not the case when using microperimetry. Moreover, the accuracy of the visual acuity assessment is often dependent on the competency level of the examiner, leading to inter-observer variability [32]. Unlike the visual acuity assessment, microperimetry is an automated functional test, meaning that the investigator does not run the same risk of acquiring unreliable data.

Microperimetry is a non-invasive procedure to assess macular sensitivity while the fundus is directly examined through live imaging [5]. Other clinical tests of visual function have been found to be useful measures in AMD. These include: contrast sensitivity [34]; dark adaptation [35] and electroretinography (ERG) [36]. Unlike in ERG, there is no standardised protocol in microperimetry. For example, no recommendations exist on whether patients should be examined with their pupils dilated or undilated, although a recent study found that patients may be tested with or without pupil dilation as both scenarios produce consistent and interchangeable results [37]. Microperimetry enables clinicians to directly relate visual function to underlying fundus morphology, giving insight into the pathophysiology and natural history of retinal disease. Even in the presence of relatively good visual acuity, such as in the early stage of AMD, microperimetry can provide relevant information regarding macular dysfunction [7, 8]. Sugawara et al. have already shown a significant positive correlation (*p* = 0.0003) between macular sensitivity as measured by microperimetry and vision-related quality of life in patients with retinitis pigmentosa [38]; now our correlations reveal that macular sensitivity relates more closely to vision-related quality of life in patients with AMD than does the ETDRS measurement of visual acuity.

Microperimetry technology contains an eye-tracking system that automatically corrects the position of the stimulus when a patient changes their fixation. The Nidek MP-3 has an eye tracking system that automatically registers the position of the eye relative to anatomical landmarks twenty-five times per second [6]. Additionally, microperimetry has been shown to have high test-retest reliability even when visual acuity is poor, and fixation is unstable and eccentric [39]. Microperimetry is therefore proven to be a useful tool in tracking disease progression when looking at treatment efficacy or performing a longitudinal study [3, 40].

The Nidek MP-3 microperimeter can provide an overall macular sensitivity by calculating the mean of all DLS points inside the region of the macula. Patients with AMD may show macular dysfunction that precedes noticeable vision loss [3, 40] and our results show that overall macular sensitivity is reduced in early and late AMD compared with eyes with healthy retina. Therefore, microperimetry may be a more sensitive screening tool for early disease than visual acuity.

In patients with AMD, DLS points can vary greatly in terms of their retinal sensitivity. This produces a wide range of results within the same macula: for example, a clinically significant difference in retinal sensitivity is found at the border of a scotoma. Analysis of retinal sensitivity at individual DLS points therefore allows for a more localised assessment of macular function [3, 7, 40] that can be helpful in the management of retinal pathology [9]. Macular subfield analysis can be used clinically in the management of patients with AMD to determine the impact of the disease on specific areas of the macula.

Although macular sensitivity is more closely aligned with vision-related quality of life than the ETDRS method of visual acuity testing, microperimetry is not without limitations. The investigation can be time-consuming and it requires good patient cooperation. Microperimetry in patients with AMD with unstable fixation can be even more time-consuming as in order for the investigation to proceed to eventual completion, eye movements must be either corrected automatically by the microperimeter or manually by the technician. Furthermore, microperimetry equipment comes at a cost to the healthcare provider [41], although we might reasonably expect the apparatus to become less expensive over time.

We chose to correlate macular subfields with the near-distance activities sub-score of the VFQ because the inability of patients with AMD to maintain steady fixation is strongly associated with slower reading [42]. This particular aspect of visual function can affect quality of life in patients with AMD [43].

In both AMD sub-groups, we observed that the nasal macula strongly correlated with VFQ composite and near activities scores. A person with healthy retina would normally use their fovea to perform near-distance activities, but patients with macular dysfunction typically recruit a parafoveal region of the macula as their preferred retinal locus for fixating and scanning text [10, 11, 44]. The strong correlation between the nasal macular sensitivity and the VFQ near activities sub-score in particular suggests that the nasal inner and outer sub-regions are preferred retinal loci in patients with early and late AMD respectively. It is known that the macula region is rich in rod photoreceptors, but that these are affected earliest and most severely in AMD [45, 46]. The highest rod densities are located along an elliptical ring at the eccentricity of the optic disc extending into the nasal macula [47], which may explain why we found this area of the macula to be useful in patients with AMD. Furthermore, rod function has been shown to be important to vision-related quality of life in patients with AMD undergoing cataract surgery [48]. Our results may therefore be suggestive of a pattern of disease-mediated rod photoreceptor loss, but further studies are required to investigate the possible clinical implications of this.

There were no statistically significant correlations observed in the group without vitreoretinal disease. This may be due to a ceiling-effect caused by a narrow range of retinal sensitivities among those without vitreoretinal disease. Indeed, a similar trend was observed by Barboni et al. in the control group of their study [40].

AMD is a heterogenous disease and a strength of this study is that we divided patients by stage: those with neovascular AMD were allocated one sub-group, while those with non-neovascular AMD without geographic atrophy were allocated another. A limitation of this study is that we did not include a sub-group of patients with geographic atrophy so that we could correlate functional outcome measures with lesion size. Future studies could seek to correlate retinal function with disease morphology in these patients. Our study did not divide the patients according to severity or phenotype: future studies could repeat our experiment with the addition of an intermediate AMD sub-group. Furthermore, in this study the AMD group and the healthy retina group were not age matched. This is an inherent difficulty in case-control studies involving AMD, as it is estimated that up to one third of the population older than 60 years have drusen clinically, and perhaps all elderly people have drusen histologically [49].

## Conclusions

Our findings show that macular sensitivity as measured using microperimetry correlates with vision-related quality of life both in patients with early AMD and in patients with late AMD. Microperimetry is a useful measure of visual outcome and we therefore recommend the implementation of microperimetry in clinical practice in order to improve the management of these patients. Use of microperimetry can also potentiate further studies that aim to investigate macular morphology and function in greater detail. In the future it may be possible to use microperimetry as a prognostic tool for predicting vision-related quality of life in patients with AMD by analysing areas of reduced macular sensitivity.

## Supplementary Information

**Additional file 1.** Word.docx; Patient characteristics; table.

**Additional file 2.** Word.docx; Median macular sensitivities in age-related macular degeneration and healthy retina using right eye as standard; table.

**Additional file 3.** Word.docx; Visual Function Questionnaire sub-scores correlated with macular sensitivities by sub-region; table.

**Additional file 4.** Word.doc; Consolidated criteria for reporting qualitative studies checklist; table.

## Data Availability

The datasets used and analysed during the current study are available from the corresponding author on reasonable request.

## Abbreviations

AMD: Age-related macular degeneration
BCVA: Best-corrected visual acuity
DLS: Differential light sensitivity
ERG: Electroretinography
ETDRS: Early Treatment Diabetic Retinopathy Study
IQR: interquartile range
OCT: Optical coherence tomography
VFQ: Visual function questionnaire
