# Supplementary material for: Correlation of macular sensitivity measures and visual acuity to vision-related quality of life in patients with age-related macular degeneration
Source: BMC Ophthalmol. 2021 Mar 23;21:149. doi: 10.1186/s12886-021-01901-x (PMC7988949; doi:10.1186/s12886-021-01901-x)
Supplement: Supplementary file 1 — Additional file 1. Word.docx; Patient characteristics; table. [file 12886_2021_1901_MOESM1_ESM.docx]

**Additional file 1:** Patient characteristics

|  | Early AMD Group | Late AMD Group | Healthy Retina Group | p values | | |
| --- | --- | --- | --- | --- | --- | --- |
|  |  |  |  | **p^1^** | **p^2^** | **p^3^** |
| Participants  (N) | 15 | 28 | 32 | - | - | - |
| Mean age  (years) | 77.5  (SD: 7.2) | 79.1  (SD: 5.3) | 71.7  (SD: 7.8) | 0.025* | <0.001* | 0.343* |
| Sex  (males / females) | 8 / 7 | 8 / 20 | 12 / 20 | 0.098† | 0.267† | 0.011† |

*Independent samples test

†Chi-squared test

AMD=age-related macular degeneration; p^1^=early AMD compared with healthy retina; p^2^= late AMD compared with healthy retina; p^3^=early AMD compared with late AMD
