# Supplementary material for: Correlation of macular sensitivity measures and visual acuity to vision-related quality of life in patients with age-related macular degeneration
Source: BMC Ophthalmol. 2021 Mar 23;21:149. doi: 10.1186/s12886-021-01901-x (PMC7988949; doi:10.1186/s12886-021-01901-x)
Supplement: Supplementary file 2 — Additional file 2. Word.docx; Median macular sensitivities in age-related macular degeneration and healthy retina using right eye as standard; table. [file 12886_2021_1901_MOESM2_ESM.docx]

**Additional file 2:** Median macular sensitivities in age-related macular degeneration and healthy retina using right eye as standard

|  | **Early AMD Sub-group** | **Late AMD Sub-group** | **Healthy Retina Group** | **p value*** |
| --- | --- | --- | --- | --- |
| Overall Macula | 21.9  IQR: 8.8 | 18  IQR: 10 | 22.61  IQR: 7.0 | <0.001 |
| Inner Ring | 20.8  IQR: 6.2 | 16.7  IQR: 9 | 22.94  IQR: 8.0 | <0.001 |
| Outer Ring | 22.3  IQR: 10.1 | 19.9  IQR: 10 | 23.13  IQR: 6.0 | 0.002 |
| Fovea | 20.0  IQR: 8.0 | 17  IQR: 8 | 23.0  IQR: 8.0 | <0.001 |
| Nasal Inner | 23.0  IQR: 5.0 | 16.7  IQR: 8 | 23.34  IQR: 6.0 | <0.001 |
| Inferior Inner | 20.3  IQR: 10.6 | 17.8  IQR: 16 | 22.67  IQR: 8.0 | 0.003 |
| Temporal Inner | 21.0  IQR: 6.0 | 18.2  IQR: 13.0 | 23.0  IQR: 7.0 | <0.001 |
| Superior Inner | 20.3  IQR: 6.0 | 15  IQR: 12 | 22.33  IQR: 8.0 | 0.001 |
| Superior Outer | 22.3  IQR: 9.0 | 20.3  IQR: 8 | 22.75  IQR: 7.0 | 0.011 |
| Nasal Outer | 21.75  IQR: 7.75 | 18.1  10 | 23.25  IQR: 6.0 | 0.001 |
| Inferior Outer | 21.5  IQR: 7.0 | 17.8  IQR: 11 | 23.0  IQR: 4.0 | <0.001 |
| Temporal Outer | 23.0  IQR: 7.0 | 22.8  IQR: 8 | 23.59  IQR: 7.0 | 0.012 |

All macular sensitivities given in decibels

*Mann-Whitney U-test

AMD=age-related macular degeneration; IQR=interquartile range
