# Supplementary material for: Correlation of macular sensitivity measures and visual acuity to vision-related quality of life in patients with age-related macular degeneration
Source: BMC Ophthalmol. 2021 Mar 23;21:149. doi: 10.1186/s12886-021-01901-x (PMC7988949; doi:10.1186/s12886-021-01901-x)
Supplement: Supplementary file 3 — Additional file 3. Word.docx; Visual Function Questionnaire sub-scores correlated with macular sensitivities by sub-region; table. [file 12886_2021_1901_MOESM3_ESM.docx]

**Additional file 3:** Visual Function Questionnaire sub-scores correlated with macular sensitivities by sub-region

| **Group** | **VFQ sub-score** | **ETDRS macular sub-region** | | | | | | | | | | | |
| --- | --- | --- | --- | --- | --- | --- | --- | --- | --- | --- | --- | --- | --- |
|  |  | **Overall** | **Inner** | **Outer** | **Fovea** | **NI** | **II** | **TI** | **SI** | **SO** | **NO** | **IO** | **TO** |
| Early AMD | Composite | 0.542†  p=0.037 | 0.114  p=0.685 | 0.211  p=0.45 | 0.15  p=0.595 | 0.501  p=0.057 | 0.045  p=0.869 | -0.02  p=0.944 | 0.246  p=0.377 | 0.105  p=0.708 | 0.482  p=0.069 | 0.1  p=0.723 | 0.111  p=0.694 |
|  | Near-distance activities | 0.595†  p=0.019 | 0.619†  p=0.014 | 0.657*  p=0.008 | 0.505  p=0.055 | 0.323  p=0.241 | 0.631†  p=0.012 | 0.577†  p=0.024 | 0.512  p=0.051 | 0.523†  p=0.045 | 0.653*  p=0.008 | 0.666*  p=0.007 | 0.464  p=0.82 |
| Late AMD | Composite | 0.458†  p=0.014 | 0.392†  p=0.039 | 0.311  p=0.107 | 0.422†  p=0.025 | 0.508†  p=0.06 | 0.43†  p=0.022 | 0.32  p=0.097 | 0.225  p=0.25 | 0.243  p=0.212 | 0.469†  p=0.012 | 0.348  p=0.069 | 0.359  p=0.06 |
|  | Near-distance activities | 0.598 *  p=<0.001 | 0.379†  p=0.047 | 0.310  p=0.108 | 0.427†  p=0.024 | 0.652*  p=<0.001 | 0.399†  p=0.036 | 0.303  p=0.117 | 0.351†  p=0.045 | 0.226  p=0.248 | 0.602  p=<0.001 | 0.369  p=0.053 | 0.177  p=0.367 |
| Healthy retina | Composite | 0.219  p=0.228 | 0.035  p=0.85 | -0.049  p=0.794 | 0.036  p=0.847 | -0.022  p=0.905 | 0.032  p=0.864 | 0.109  p=0.561 | 0.05  p=0.789 | 0.151  p=0.418 | 0.275  p=0.128 | -0.034  p=0.857 | -0.09  p=0.625 |
|  | Near-distance activities | 0.151  p=0.41 | 0.193  p=0.299 | 0.096  p=0.608 | 0.221  p=0.232 | 0.023  p=0.899 | 0.153  p=0.411 | 0.234  p=0.205 | 0.223  p=0.229 | 0.258  p=0.161 | 0.104  p=0.570 | -0.017  p=0.929 | 0.124  p=0.499 |

Overall, NI and NO correlations are Pearson’s *r*. All other correlations are Spearman’s *rho*

*indicates correlation is significant at the 0.01 level (2-tailed).

†indicates correlation is significant at the 0.05 level (2-tailed).

AMD=age-related macular degeneration; ETDRS=Early Treatment Diabetic Retinopathy Study; II=inferior inner; IO=inferior outer; NI=nasal inner; NO=nasal outer; SI=superior inner; SO=superior outer; TI=temporal inner; TO=temporal outer; VFQ=Visual Function Questionnaire
